# Inter-reader agreement of the BI-RADS CEM lexicon

**DOI:** 10.1007/s00330-024-11176-7

**Published:** 2024-11-06

**Authors:** Calogero Zarcaro, Ambra Santonocito, Layla Zeitouni, Francesca Ferrara, Panagiotis Kapetas, Ruxandra-Iulia Milos, Thomas H. Helbich, Pascal A. T. Baltzer, Paola Clauser

**Affiliations:** 1 Department of Biomedicine, Neuroscience and Advanced Diagnostic (Bi.N.D.), University Hospital “Policlinico P. Giaccone”, Palermo, Italy; 2 Department of Biomedical Imaging and Image-Guided Therapy, Medical University of Vienna, Vienna, Austria; 3 Department of Radiology Section of Breast Imaging, King Faisal Specialist Hospital and Research center, Riyadh, Saudi Arabia; 4 Catholic University of the Sacred Heart, Insitute of Radiology, Largo A, Rome, Italy; 5 Department of Radiology, Breast Imaging Service, Memorial Sloan Kettering Cancer Center, New York, NY USA

**Keywords:** Mammography, Contrast media, Observer variation, Breast neoplasms

## Abstract

**Purpose:**

The purpose of this study was to assess the inter-reader agreement of the breast imaging reporting and data system (BI-RADS) contrast-enhanced mammography (CEM) lexicon.

**Materials and methods:**

In this IRB-approved, single-center, retrospective study, three breast radiologists, each with different levels of experience, reviewed 462 lesions in 421 routine clinical CEM according to the fifth edition of the BI-RADS lexicon for mammography and to the first version of the BI-RADS lexicon for CEM. Readers were blinded to patient outcomes and evaluated breast and lesion features on low-energy (LE) images (breast density, type of lesion, associated architectural distortion), lesion features on recombined (RC) images (type of enhancement, characteristic of mass enhancement, non-mass enhancement or enhancing asymmetry), and provided a final BI-RADS assessment. The inter-reader agreement was calculated for each evaluated feature using Fleiss’ kappa coefficient. Sensitivity and specificity were calculated.

**Results:**

The inter-reader agreement was moderate to substantial for breast density (ĸ = 0.569), type of lesion on LE images (ĸ = 0.654), and type of enhancement (ĸ = 0.664). There was a moderate to substantial agreement on CEM mass enhancement descriptors. The agreement was fair to moderate for non-mass enhancement and enhancing asymmetry descriptors. Inter-reader agreement for LE and LE with RC BI-RADS assessment was moderate (ĸ = 0.421) and fair (ĸ = 0.364). Diagnostic performance was good and comparable for all readers.

**Conclusion:**

Inter-reader agreement of the CEM lexicon was moderate to substantial for most features. There was a low agreement for some RC descriptors, such as non-mass enhancement and enhancing asymmetry, and BI-RADS assessment, but this did not impact the diagnostic performance.

**Key Points:**

***Question*** *Data on the reproducibility and inter-reader agreement for the first version of the BI-RADS lexicon dedicated to CEM are missing*.

***Finding*** *The inter-reader agreement for the lexicon was overall substantial to moderate, but it was lower for the descriptors for non-mass enhancement and enhancing asymmetry*.

***Clinical relevance*** *A common lexicon simplifies communication between specialists in clinical practice. The good inter-reader agreement confirms the effectiveness of the CEM-BIRADS in ensuring consistent communication. Detailed definitions of some descriptors (non-mass, enhancing asymmetry) are needed to ensure higher agreements*.

## Introduction

The American College of Radiology Breast Imaging Reporting and Data System was developed with the aim of establishing a consistent and standardized approach for reporting breast imaging findings. Initially introduced in 1992, the first edition of the BI-RADS Atlas focused exclusively on mammography (MG). Subsequently, the fifth edition of the BI-RADS atlas was published in 2013, which further refined the terms and updated the lexicon for MG, ultrasound (US), and magnetic resonance imaging (MRI) [1].

Contrast-enhanced mammography (CEM) is an emerging technique that, using a dual-energy image acquisition and iodinated contrast media, provides low-energy (LE) 2D mammographic images, comparable to standard MG [2], and post-contrast recombined (RC) images, which allow the visualization of tumor neovascularization, similar to MRI [3]. Several studies have shown the increased sensitivity and specificity of CEM compared to MG [4–6] and a diagnostic performance similar to that of MRI [7, 8] for a wide variety of clinical applications. Since CEM received Food and Drug Administration approval in 2011, its use has increased exponentially. While the interpretation of CEM images has some obvious overlap with MG and MRI, it is also characterized by some method-specific findings. Thus, in 2022, the first version of the BI-RADS lexicon for CEM was published as a supplement to the fifth edition of the BI-RADS MG lexicon [9], with the aim of introducing a standardized terminology and achieving greater consistency and accuracy in reporting. To facilitate their use and adoption in clinical practice, the descriptors chosen have similarities with the BI-RADS MG and MRI lexicon.

To date, only a limited number of studies have been performed using this new lexicon, and its applicability in clinical practice has not been widely investigated. The aim of our study was to assess the inter-reader agreement of breast imagers using the first edition of the BI-RADS lexicon to interpret breast CEM.

## Materials and methods

### Study design and patient population

This retrospective, single-center study received approval from the Institutional Review Board, and the need for informed consent was waived.

Included were women who underwent CEM for inconclusive findings on conventional imaging, classified as BI-RADS 0, or who were recalled from screening for the presence of suspicious findings. Exclusion criteria were incomplete CEM examination, lack of a visible lesion on CEM, image-guided biopsy performed before CEM, and lack of a standard of reference.

When available, histology was considered as a standard of reference. Image-guided biopsies were performed using US, tomosynthesis, stereotactic, or MRI guidance after the acquisition of CEM. When histology was not available, an image follow-up of at least 12 months was considered. Finally, 462 lesions visible in LE images, RC images, or both were included in the analysis, from 421 women (age range: 30–90 years; mean age ± standard deviation: 54.3 ± 10.8 years) who underwent CEM between October 2018 and September 2022.

### CEM acquisition protocols

CEM was acquired using a Siemens Mammomat Revelation unit (Siemens Healthineers, Erlangen, Germany). To obtain the images, a sequential approach was used, with a dual-energy examination carried out after intravenous injection of contrast medium, during a single breast compression. A single dose of 1 mL/kg body weight of non-ionic iodinated contrast agent (Iomeron® 400, Bracco) was given at a rate of 3 mL/s using a power injector (Ulrich Medical), followed by a flush of 20 mL of saline. Sixty to 120 s after injecting the contrast agent, image acquisition was started from the breast with suspicious or unclear findings.

The first image was obtained with low energy (LE), which is comparable to a standard digital MG. Subsequently, during the same compression, the high energy (HE) image is acquired, focused on capturing iodine information, and maximizing the dual-energy contrast. The HE images were obtained using a tungsten anode and a 1-mm titanium filter, set at a fixed tube voltage of 49 kVp, while an additional titanium filter was used. Conversely, the LE images were taken using a tungsten anode target and a 55-μm rhodium filter at a tube voltage of 26–32 kVp, following standard mammographic imaging protocols that use photon energies below the K-edge of iodine. Both energy exposures were obtained using an automatic image acquisition technique, while the LE images adhered to standard mammogram acquisition parameters to ensure consistent image acquisition and processing settings. Finally, post-processed RC images were generated from the low- and high-energy images.

### CEM image interpretation

Three radiologists, each from different institutions and with different levels of experience in CEM assessment (1–3 years), assessed the CEM images. Readings were performed in separate sessions, and readers were blinded to histological results and patient history, using the fifth edition of the BI-RADS MG lexicon [10] and the first edition of the BI-RADS CEM lexicon [9]. The readers were aware of the location of the lesion (side, quadrant) to ensure the evaluation of the same lesion. Readings were performed on dedicated high-resolution monitors (12 MP Monitor, 30 BIT, Pixel pitch: 0.1686 mm, Coronis unit, BarcoMed).

For each lesion, the readers were asked to define:

- On LE images, ACR breast density (almost entirely fatty, scattered areas of fibroglandular tissue, heterogeneously dense, and extremely dense); presence/absence of a lesion; if present, the type of lesion (mass, microcalcifications, asymmetry, and define if more than one lesion); presence/absence of associated architectural distortion.
- On RC images, presence/absence of enhancement; if present, the type of enhancement (mass, non-mass, and enhancing asymmetry); lesion conspicuity (low, moderate, and high). For mass enhancement, shape (oval, round, and irregular), margin (circumscribed, irregular, and spiculated), and internal enhancement characteristics (homogeneous, heterogeneous, and rim enhancement) were recorded. Regarding non-mass enhancement, the recorded features included distribution (focal, linear, segmental, regional, multiple regions, and diffuse) and internal enhancement patterns (homogeneous, heterogeneous, and clumped). The evaluation of enhancing asymmetry focused on the internal pattern of enhancement (homogeneous and heterogeneous).
- A BI-RADS score for each lesion was assigned using categories 2–5. The CEM evaluation was carried out considering both LE and RC images; for example, in the case of suspicious microcalcifications classified as BI-RADS 4 on LE images, even when no enhancement was visible in the RC images, the final assessment was BIRADS 4, and not BI-RADS 1.

Maximum lesion diameter considering LE and RC images together was also recorded.

### Statistical analysis

The inter-reader agreement was calculated for each breast and lesion feature (e.g., mass margin), for each individual descriptor (e.g., mass margin circumscribed), and for BI-RADS assessments (LE and CEM) using unweighted Fleiss’ kappa coefficient [11] for evaluation among three interpreters. According to a commonly used scale, a ĸ of 0.2 or lower indicates slight agreement; 0.21–0.40 indicates fair agreement; 0.41–0.60 indicates moderate agreement; 0.61–0.80 indicates substantial agreement; and 0.81–0.99 indicates almost perfect agreement [12]. Finally, an analysis of the diagnostic performance (sensitivity and specificity) of the three readers was carried out, considering benign lesions classified as BI-RADS 1–3 and malignant those classified as 4 and 5. Statistical analysis was performed with SPSS Statistics (version 29.0.1.0, IBM).

## Results

The lesions included in the analysis (size range: 3–100 mm; mean size ± SD: 16.55 ± 18.06 mm) were originally interpreted as follows: on LE images, 154 were mass lesions, 121 microcalcifications only, 78 were asymmetries, while in the remaining cases, no findings were visible. In 28 patients, more than one finding was present. On RC images findings were: mass enhancement in 150 cases, non-mass enhancement in 90 cases, enhancing asymmetries in 37 cases, while in 185 cases no post-contrast findings were visible. According to the standard of reference, 302 (65.4%) lesions were benign and 160 (34.6%) were malignant.

### LE images

**Fig. 1:**
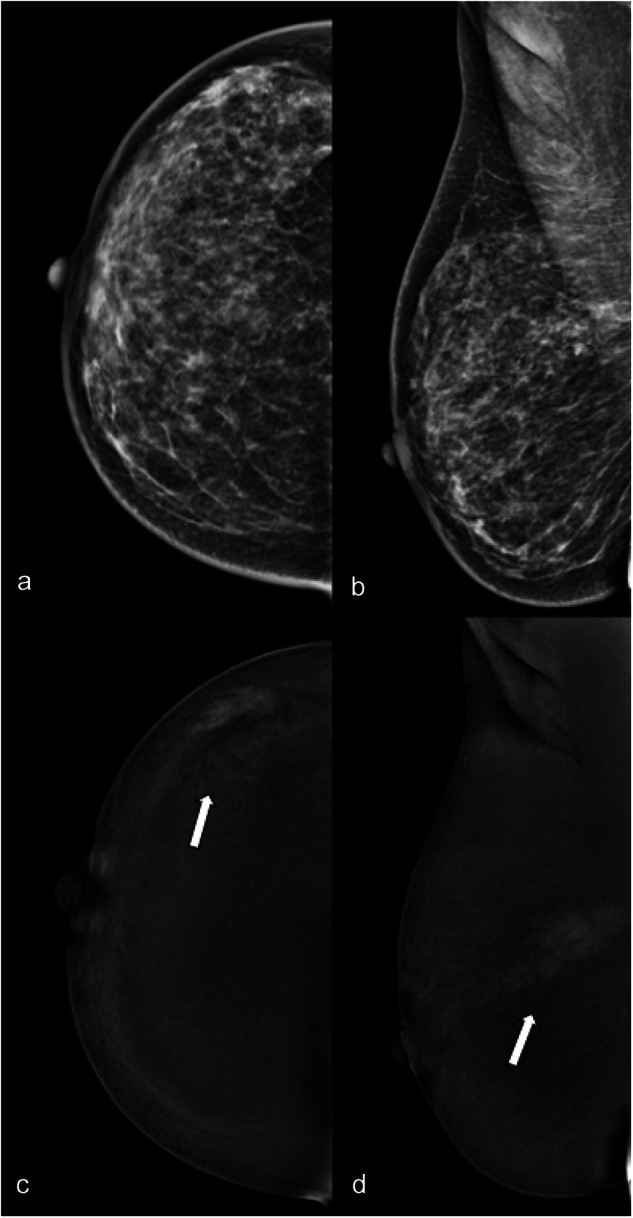
LE (**a**, **b**) and RC (**c**, **d**) CEM images of the right breast of a 32-year-old patient with nipple discharge. The three readers evaluated in separate sessions ACR density (**c**, unanimously); type of lesion (microcalcifications for two of the three readers, mixed for the third); presence/absence of associated architectural distortion (absence, unanimously) on LE images; type of enhancement (white arrows: non-mass, unanimously); lesion conspicuity (high, unanimously) and non-mass enhancement descriptors (non-mass distribution segmental, unanimously; and the non-mass internal pattern of enhancement heterogeneous for two of the three readers, clumped for the third) on RC images. The level of suspicion on LE was BI-RADS 4, unanimously, and on RC BI-RADS 4 for two of the three readers and 5 for the third. Histology was ductal carcinoma in situ with micro invasion

The overall agreement between the three readers was moderate for breast density and substantial for lesion type on LE images (Table 1). There was an almost perfect agreement in the identification of microcalcifications (ĸ = 0.820) (Fig. 1). The inter-reader agreement for the identification of architectural distortion was moderate (ĸ = 0.496).

**Table 1:** Inter-reader agreement for breast density and lesion features on LE CEM images

Feature	Three-reader *k*
ACR breast density	0.569
Almost entirely fatty	0.515
Scattered areas of fibroglandular density	0.601
Heterogeneously dense	0.540
Extremely dense	0.596
MG type of findings	0.654
No lesion	0.674
Mass	0.624
Calcifications	0.820
Asymmetry	0.529
More than one lesion	0.498
Architectural distortion associated	0.496
Absent	0.496
Present	0.496

*ACR* American College of Radiology, *MG* mammography

### RC images

**Fig. 2:**
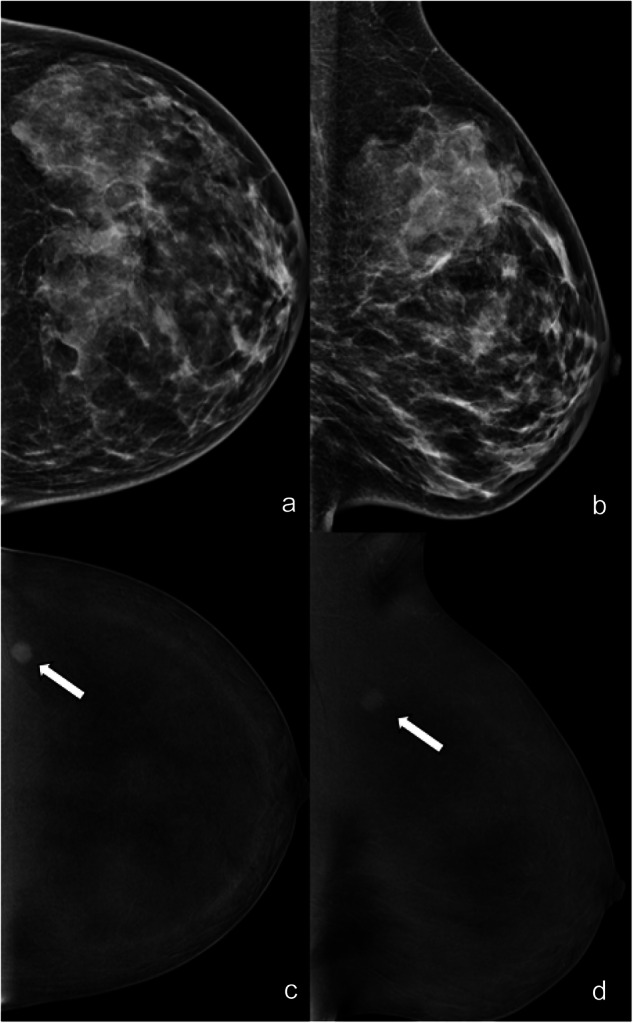
LE (**a**, **b**) and RC (**c**, **d**) CEM images of the left breast of a 35-year-old patient. The three readers evaluated in separate sessions ACR density (**d**, unanimously); type of lesion (mass for two of the three readers, asymmetry for the third); presence/absence of associated architectural distortion (absence and unanimously) on LE images; type of enhancement (white arrows: mass, unanimously); and lesion conspicuity (high for two of the three readers, moderate for the third) and mass enhancement descriptors (mass shape round, mass margin irregular, mass internal pattern of enhancement homogeneous, unanimously) on RC images. Finally, they assessed LE BI-RADS (4 for two of the three readers, 3 for the third) and RC BI-RADS (5 for two of the three readers, 4 for the third). Histology was triple-negative breast cancer

**Fig. 3:**
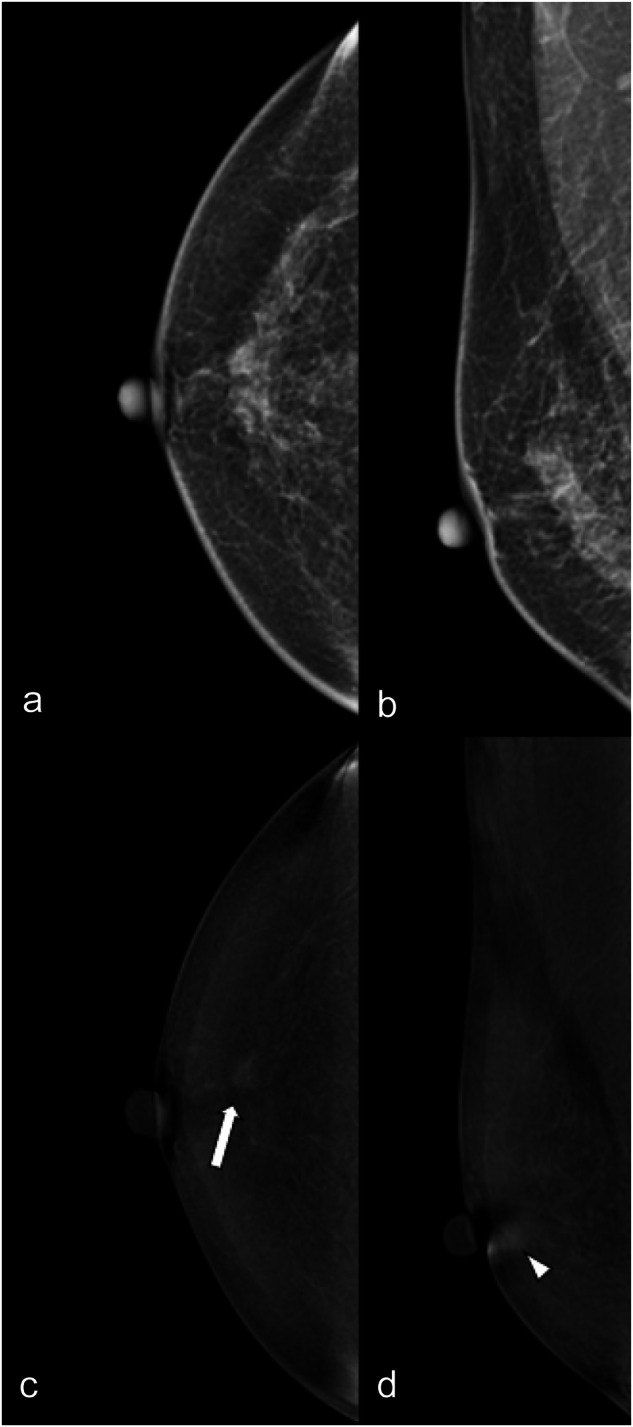
LE (**a**, **b**) and RC (**c**, **d**) CEM images of the right breast of a 69-year-old patient. The three readers evaluated in separate sessions ACR density (**b**, unanimously); type of lesion (no lesion for two of the three readers, mixed for the third); presence/absence of associated architectural distortion (absence, unanimously) on LE images; type of enhancement (enhancing asymmetry for two of the three readers, evidenced by white arrows on CC view; non-mass for the third, evidenced by white arrowhead on MLO view); and lesion conspicuity (moderate for two of the three readers, low for the third) and mass enhancement descriptors (enhancing asymmetry internal pattern of enhancement homogeneous for two of the three readers, non-mass distribution focal and non-mass internal pattern of enhancement homogeneous for the third) on RC images. The level of suspicion was BI-RADS 1 for two of the three readers and for the third on LE and on RC BI-RADS 4, unanimously. Histology after US-guided biopsy was atypical ductal hyperplasia, final diagnosis at surgery was ductal carcinoma in situ

The inter-agreement was substantial for the type of enhancement on RC images (no enhancement, mass, non-mass, enhancing asymmetry) (ĸ = 0.664, Table 2). There was a moderate agreement for lesion conspicuity (ĸ = 0.517). The overall agreement for mass enhancement descriptors was moderate for shape (ĸ = 0.523) and margins (ĸ = 0.566) and substantial for an internal pattern of enhancement (ĸ = 0.618) (Fig. 2). The agreement for non-mass enhancement descriptors was fair for distribution (ĸ = 0.387) and moderate for an internal pattern of enhancement (ĸ = 0.415). The agreement for enhancing asymmetry descriptors was fair (ĸ = 0.247) (Fig. 3).

**Table 2:** Inter-reader agreement for lesion features on RC CEM images

Feature	Three-reader *k*
CEM type of enhancement	0.664
No enhancement	0.791
Mass	0.733
Non-mass	0.541
Enhancing asymmetry	0.320
CEM lesion conspicuity	0.517
Low	0.566
Moderate	0.392
High	0.586
Mass shape	0.523
Oval	0.392
Round	0.494
Irregular	0.375
Mass margin	0.566
Circumscribed	0.465
Irregular	0.470
Spiculated	0.602
Mass internal enhancement pattern	0.618
Homogeneous	0.407
Heterogeneous	0.687
Rim enhancement	0.409
Non-mass distribution	0.387
Focal	0.265
Linear	0.205
Segmental	0.416
Regional	0.158
Multiple regions	0.000*
Diffuse	0.273
Non-mass internal enhancement pattern	0.417
Homogeneous	0.122
Heterogeneous	0.473
Clumped	0.286
Enhancing asymmetry pattern	0.247
Homogeneous	0.175
Heterogeneous	0.180

* Only two readers used the non-mass enhancement distribution descriptor “multiple regions”, for two different cases

### Benign and malignant lesions

**Fig. 4:**
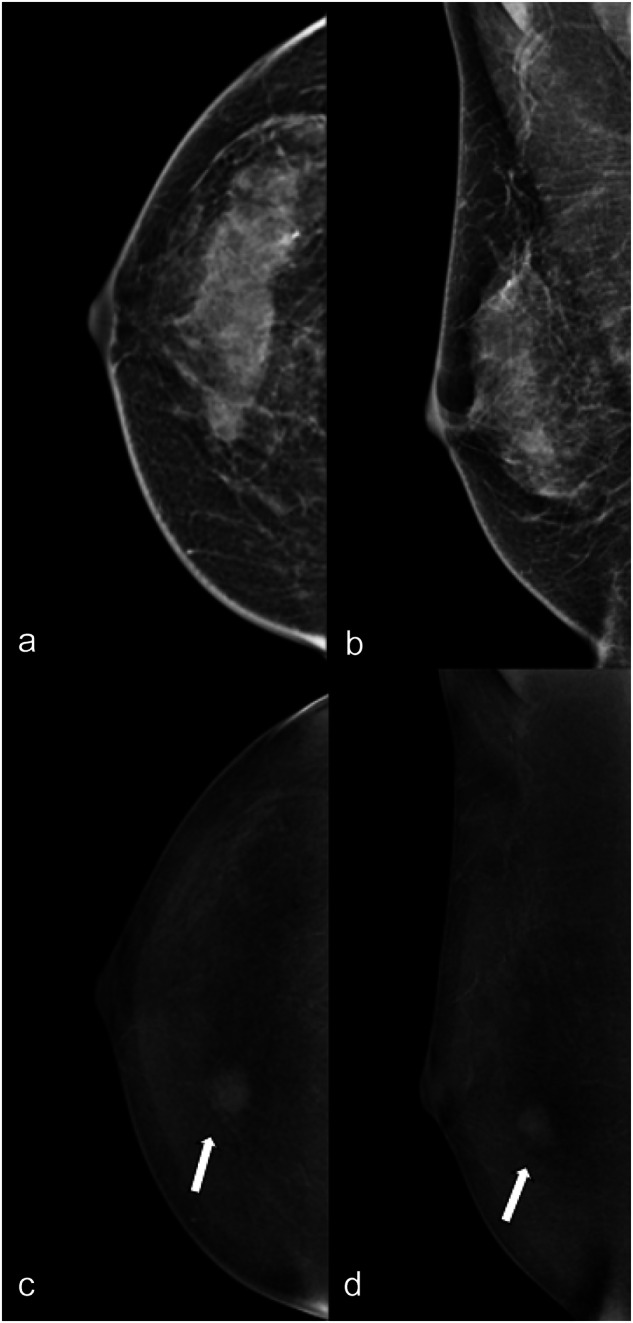
LE (**a**, **b**) and RC (**c**, **d**) CEM images of the right breast of a 49-year-old patient. The three readers evaluated in separate sessions ACR density (**c**, unanimously); type of lesion (mass for two of the three readers, no lesion for the third); presence/absence of associated architectural distortion (absence, unanimously) on LE images; type of enhancement (white arrows: mass, unanimously); lesion conspicuity (high for two of the three readers, moderate for the third) and mass enhancement descriptors (mass shape round for two of the three readers, oval for the third; mass margin circumscribed for two of the three readers, irregular for the third; the mass internal pattern of enhancement heterogeneous for two of the three readers, homogeneous for the third; and mass internal pattern of enhancement heterogeneous for two of the three readers, homogeneous for the third) on RC images. Finally, they assessed a LE BI-RADS (4 for two of the three readers, 1 for the third) and RC BI-RADS (4, unanimously). Histology was invasive ductal carcinoma

Table 3 shows the agreement values among all three readers considering benign and malignant lesions separately. Agreement on the non-mass descriptors (distribution and internal enhancement pattern) was substantially higher for malignant lesions (moderate, 0.423 and 0.432, respectively) than for benign lesions (Fig. 4). In contrast, agreement on lesion conspicuity was substantially lower for malignant lesions (fair, *k* = 0.357) than for benign lesions (0.535).

**Table 3:** Inter-reader agreement on breast lesions features for benign and malignant lesions separately

Feature	Benign (302), *k*	Malignant (160), *k*
LE type of lesion	0.664	0.612
Architectural distortion associated	0.486	0.442
CEM type of enhancement	0.624	0.617
CEM lesion conspicuity	0.535	0.357
Mass shape	0.507	0.467
Mass margin	0.527	0.531
Mass internal enhancement pattern	0.554	0.588
Non-mass distribution	0.328	0.423
Non-mass internal enhancement pattern	0.373	0.432
Enhancing asymmetry pattern	0.236	0.268

### BI-RADS assessment and diagnostic performance

Inter-reader agreement among all three readers for LE and CEM (RC with LE images) BI-RADS assessment was moderate (ĸ = 0.421) and fair (ĸ = 0.364).

Despite the low agreement, sensitivity was high and improved with CEM for all three readers. Specificity varied between readers with CEM (Table 4).

**Table 4:** Sensitivity and specificity of the readers evaluating the examinations using BI-RADS on LE images and on both LE and RC images together (CEM)

	R1	95% CI R1	R2	95% CI R2	R3	95% CI R3	*p*-value
LE BIRADS sensitivity	77%	0.705–0.835	78%	0.716–0.844	74%	0.672–0.808	< 0.001
LE BIRADS specificity	67%	0.617–0.723	75%	0.701–0.799	70%	0.648–0.751	< 0.001
CEM BIRADS sensitivity	88%	0.830–0.930	90%	0.854–0.946	91%	0.866–0.954	< 0.001
CEM BIRADS specificity	67%	0.617–0.723	80%	0.755–0.845	52%	0.464–0.576	< 0.001

*R* reader, *CI* confidence interval

## Discussion

The CEM BI-RADS lexicon provides standardized and formal definitions for the description of breast lesions and facilitates communication between breast specialists [9, 10]. Its clinical utility depends on the ability of individual readers to agree on interpretations, to ensure consistency in clinical recommendations. In our experience, inter-reader agreement on the CEM lexicon was moderate-substantial for most features, with some exceptions for RC descriptors and BI-RADS assessment, which did not affect diagnostic performance.

Focusing on RC images only, the strongest agreement was found for the assessment of the type of enhancement, particularly for the identification of masses (ĸ = 0.73). The inter-reader agreement was moderate for non-masses, while it was only fair for the definition of enhancing asymmetry. To the best of our knowledge, this is the first study to assess inter-reader agreement for the CEM BI-RADS lexicon. Therefore, our results about post-contrast features can be compared only with previous studies that focused on MRI, particularly the study by Grimm et al [13]. The agreement on the assessment of the type of enhancement obtained in their study on MRI is lower than that obtained from our study on CEM; these findings could be related to the reader’s greater familiarity with these CEM descriptors as they are similar to those used in breast MRI. Considering the descriptors separately, however, the trend of the results reverses, with the agreement for each descriptor separately being slightly higher in the study by Grimm et al on MRI [13]. In our analysis, the highest agreement was found for mass enhancement, namely moderate on shape and margin, and substantial on internal enhancement pattern. These levels of agreement are slightly lower than those found for MRI, which were substantial for all three mass descriptors [13]. The agreement on CEM non-mass descriptors was overall lower, especially regarding non-mass distribution. The study by Grimm et al [13] found a substantial agreement, despite the fact that the descriptors provided in CEM evaluation were the same as those used in MRI. As is already known for MRI, non-mass lesions are more complex to detect and characterize [14, 15]; this seems to be true also for CEM, and to be reflected by a lower inter-reader agreement for CEM, in which these difficulties are increased by the lack of 3D data.

A comparison between CEM and MRI was made in the study of Knogler et al [16], who, before the release of the CEM BI-RADS lexicon, used the MRI BI-RADS descriptors for the evaluation of CEM images. They demonstrated that enhancement characteristics were similar in the malignant cases, findings confirmed by our study in the analysis considering benign and malignant lesions separately.

The lowest agreement was found for the definition of enhancing asymmetry. As this descriptor was not present in previous imaging modalities and it is a newly introduced term with which readers are still unfamiliar, this might explain the results. Another aspect that might justify the low agreement for the descriptors of non-mass enhancement and enhancing asymmetry is the difference between two “similar” terms belonging, however, to different breast imaging lexicons, namely asymmetries in the BI-RADS MG lexicon [10] and enhancing asymmetry in the BI-RADS CEM lexicon [9]. In the BI-RADS MG lexicon, the term asymmetry refers to an area of fibro glandular-dense tissue, not conforming to the definition of a radiodense mass, that is visible on only one mammographic projection. In addition to asymmetry, however, there are three other types of asymmetries in the same lexicon, namely global asymmetry, focal asymmetry, and developing asymmetry which are visible in more than one projection. This difference in the spectrum of asymmetries is not present in the CEM BI-RADS lexicon and may have led to confusion in the interpretation of the findings. For example, an enhancement that cannot be defined as a mass, visible in more than one RC projection, should not be defined as an “enhancing asymmetry” but as a “non-mass enhancement with focal distribution”. The low agreement values on the non-mass enhancement and enhancing asymmetry descriptors suggest that they need to be evaluated further in the future.

In 2022, Nicosia et al [17] created a predictive score for the malignancy of a breast lesion based on the main contrast enhancement features (intensity, pattern, margin, and ground glass) on CEM; an experienced breast imaging radiologist was asked to evaluate 377 lesions and assign a score of 0 or 1 for each descriptor, depending on whether the enhancement characteristic was predictive of benignity or malignancy. Then, an overall enhancement score ranging from 0 to 4 was obtained and the histological results were considered the gold standard in the evaluation of the relationship between enhancement patterns and malignancy. Although the study was conducted before the publication of the BIRADS CEM lexicon, some of the descriptors they used are the same as those found in the lexicon (e.g., regular or irregular margin morphology, homogeneous or heterogeneous enhancement pattern). The study by Nicosia et al [17] showed that some features of mass and non-mass enhancements on CEM are important predictors for the differentiation of benign from malignant lesions. Inter-reader agreements for major malignancy predictors such as “irregular and spiculated mass margins” and “heterogeneous enhancement pattern” were notably high in our study, particularly among malignant lesions. Similarly, benign features such as “circumscribed mass margins” and “homogeneous mass internal enhancement pattern” showed moderate inter-reader agreement, especially among benign lesions. These findings suggest readers’ familiarity with these descriptors, particularly in malignancy characterization.

The agreement for the assessment of breast density on LE images was moderate (ĸ = 0.569), consistent with that of earlier studies that have carried out the same analysis [18–20]; specifically, in our study, the highest agreement was recorded for the category “scattered areas of fibroglandular density” (ĸ = 0.601), which, in the study by Ciatto et al [19] on DM, did not show a high agreement (ĸ = 0.25). Increased use of the BI-RADS, and consequent readers’ confidence in the classification, might explain this difference. The overall agreement for the description of the type of lesion on LE images was substantial (ĸ = 0.654). Similar results were also reported by Berg et al [18] and Lee et al [21]; in particular, in our study, there was an almost perfect agreement in the identification of microcalcifications (ĸ = 0.820), the same value reported by Lee et al [21]. Regarding the evaluation of the associated architectural distortion, the inter-reader agreement in our study was moderate (ĸ = 0.496), significantly higher than that reported by Lee et al (fair, ĸ = 0.28) [21]. In this instance, together with readers’ experience, improvements in image quality in MG in the last several years might have facilitated the detection of these types of findings. Overall, compared to previous studies conducted on DM, the interobserver agreement on LE image features was equal if not greater, particularly for associated architectural distortions.

The agreement for the final BI-RADS assessment reported in our analysis (LE BI-RADS ĸ = 0.421, CEM BI-RADS ĸ = 0.364) might seem low, but it was not dissimilar from that reported by previous studies on MG [18, 22]. The evaluation of the diagnostic performance of individual readers, with a good performance, especially for sensitivity, which was similar to that in studies from the literature [4, 5, 7, 23], confirms that these results reflect a variation in the assessment threshold of individual readers for categories with similar outcomes (biopsy for BI-RADS 4–5; no biopsy for BI-RADS 1-2-3). The lower agreement in the double evaluation pre- and post-contrast, compared with that in LE, could be related to not only the readers’ greater familiarity in scoring of non-contrast images but also to the greater variability that the findings in RC images add.

Our study has some limitations. The design of our study was retrospective and conducted in a single center, although the three readers were from different institutions and had been trained in different centers; this may have reduced their agreement, although they were all familiar with the BI-RADS lexicon. The three readers had similar levels of experience in CEM, so it was not possible to examine the potential relevance of different levels of experience, even in a two-reader analysis. Despite the fact that the readers had breast imaging experience in interpreting CEM images, the results may have been influenced by the lack of familiarity with the terminology of the CEM BI-RADS lexicon, as it is the first edition and has been released relatively recently. It is likely that the inter-reader agreement could be improved by repeating the study after a longer exposure and regular use of the new lexicon. Finally, the images were acquired on a single vendor scanner and with only one type of contrast medium; agreement might differ between different vendors.

In conclusion, the results of our study showed moderate to substantial inter-reader agreement for most lesion features on both LE and RC images. A lower inter-reader agreement was found for the descriptors of non-mass enhancement and the newly introduced enhancing asymmetry. These might be related to the lack of familiarity of the readers with the new descriptor. Inter-reader agreement for LE and CEM BI-RADS assessment was moderate and fair, respectively. Looking at the diagnostic performance, the disagreement on BI-RADS assessment did not translate into a significant variability in breast cancer diagnosis.

## Abbreviations

CEM: Contrast-enhanced mammography
HE: High energy
LE: Low energy
MG: Mammography
RC: Recombined
US: Ultrasound
